# A Groundbreaking Electric Field‐Induced Cascade Gas Therapy Against Large Volume Solid Tumor Through Electro‐Stress Storm

**DOI:** 10.1002/EXP.20240410

**Published:** 2025-12-21

**Authors:** Gui Chen, Wenjia Zhang, Manchun Wang, Fengling Zhang, Mengliang Zhu, Yan Tang, Yixian Xie, Wen Ma, Peter Timashev, Massimo Bottini, Yingqiu Xie, Xing‐Jie Liang, Meng Yu, Zhiqiang Yu

**Affiliations:** ^1^ Department of Laboratory Medicine, Dongguan Key Laboratory of Innovative Molecular Imaging, Dongguan Institute of Clinical Cancer Research, The Tenth Affiliated Hospital Southern Medical University (Dongguan People's Hospital) Dongguan P. R. China; ^2^ Department of Hepatobiliary, Pancreatic and Splenic Surgery, The Tenth Affiliated Hospital Southern Medical University (Dongguan People's Hospital) Dongguan P. R. China; ^3^ NMPA Key Laboratory for Research and Evaluation of Drug Metabolism & Guangdong Provincial Key Laboratory of New Drug Screening, School of Pharmaceutical Sciences Southern Medical University Guangzhou P. R. China; ^4^ CAS Key Laboratory for Biomedical Effects of Nanomaterials and Nanosafety, CAS Center for Excellence in Nanoscience National Center for Nanoscience and Technology of China Beijing P. R. China; ^5^ University of Chinese Academy of Sciences Beijing P. R. China; ^6^ Institute for Regenerative Medicine Sechenov University Moscow Russia; ^7^ Department of Experimental Medicine University of Rome Tor Vergata Rome Italy; ^8^ Department of Biology, School of Sciences and Humanities Nazarbayev University Astana Kazakhstan

**Keywords:** cascade gas therapy, electric field, electro‐stress storm, electrotoxicity, immunotherapy, large volume solid tumor

## Abstract

Gas therapy has been limited in its application as a robust standalone antitumor strategy due to the restricted gas production and cytotoxicity. To address this challenge, we employed electrotoxic PtRu composite metal nano‐berries (PR) loaded with various therapeutic gas donors to construct a groundbreaking electric field‐induced cascade gas therapy (EGT) platform, which generated a great electro‐stress storm at tumor sites, exerting electrotoxicity and immunity functions against solid tumors, including those of large volume, through three pathways. Initially, electric field stimulation effectively boosted the release rate and yield of therapeutic gases from the EGT platform. Further, gas molecules reacted with reactive oxygen species (ROS) to either form oxidation coordination (CO and ROS) or generate more potent therapeutic components (RNS produced from ROS and NO), contributing to an electro‐stress storm that augmented the cytotoxic potential of the gas components. Subsequently, this electro‐stress storm further activated the tumor immune response, identifying and capturing escaped tumor cells, which held significant implications for treating tumors, including non‐solid tumors with indistinct boundaries. In summary, the EGT platform leveraged an electro‐stress storm to achieve ablation of large volume solid tumors and suppressed metastatic tumors, paving new pathways for gas‐based therapeutic strategies.

## Graphical abstract

A novel electric field‐induced cascade gas therapy platform was developed to significantly enhance gas generation efficiency and cytotoxicity at the tumor site through electro‐stress storm against large volume solid tumor. The adjustable electrical stimulation contributed to the flexible spatiotemporal release and increased gas production. The gas molecules generated more potent anti‐tumor substances through oxidative reactions, provoking a powerful electro‐stress storm. More excitingly, the EGT‐based electro‐stress storm can also elicit tumor immune response in adjuvant immunotherapy.

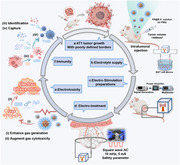

## 1 Introduction

Gas therapy has emerged as a “green” and a promising therapeutic modality in nanomedicine over the past few decades due to its negligible side‐effects and convenient deep‐tissue penetration. Generally, this strategy involves a variety of bioactive gas signal molecules such as hydrogen sulfide (H_2_S) [1], nitric oxide (NO) [2, 3], carbon monoxide (CO) [4, 5], carbon dioxide (CO_2_) [6], sulfur dioxide (SO_2_) [7, 8], and hydrogen (H_2_) [9, 10]. These molecules have been employed to regulate the homeostasis of physiological functions for various disease treatments, particularly in the field of antitumor treatment [11]. Despite its potential, the development of gas‐based clinical therapies is hindered by several challenges. The complex physiological environment of the human body poses substantial difficulties in the diffusion and delivery of gases, hindering the precise localization of lesions and the accurate control of doses. Additionally, the current level of antitumor activity exhibited by gases is regarded as insufficient. Collectively, these challenges have significantly precluded the widespread implementation of gas therapy in clinical practice [12]. To overcome these obstacles, researchers have been exploring ways to enhance the therapeutic efficacy of gas on tumors through internal and external stimuli to boost effective gas concentrations [13], and to achieve precise and targeted gas control release [14]. Among the multitude of brilliant resolutions, external electrical stimulation or photon‐driven techniques greatly stood out for their ability to bolster gas productivity with adjustable parameters in a temporally and spatially controlled manner [15, 16, 17, 18].

Despite the fact that electrical stimulation and response systems can effectively increase the efficiency of gas delivery and precise release [19, 20, 21], however, the low toxicity and low efficacy of gas therapy make it difficult to achieve the elimination of tumors. Therefore, it is often used as an adjuvant antitumor therapy [21, 22, 23, 24]. Moreover, on account of heterogeneity and immune escape of tumors, they are commonly prone to recurrence after monotherapy with gas or other drugs [25, 26]. Therefore, the emergence of combination therapies is imperative not only to improve the clinical utilization of gas therapy but also to overcome the difficulties of tumor treatment [27, 28, 29]. A groundbreaking electric field‐induced cascade gas therapy (EGT) platform has emerged to greatly boost the efficiency of gas therapy while enabling precisely controlled gas release via flexible electrical field conditions. The catalytic action of metallic nanoparticles and electrical stimulation can synergistically elevate the yield of cytotoxic substances [30]. Moreover, the electrolysis effect during EGT catalyzes the generation of reactive oxygen species (ROS) to further react with gas molecules to form more efficient therapeutic agents with longer half‐life and greater cytotoxicity (e.g., reactive nitrogen species, RNS, obtained from reaction between ROS and NO) [31, 32], leading to severe electro‐stress storm at the tumor site, finally providing deadly lethality to tumors, especially the relatively large‐sized tumor that lack of efficient ablation strategies [33].

The EGT, as a monotherapy approach, is a functional therapeutic tool with electrotoxicity property, enabling efficient ablation of relatively large tumors which is considered to be a middle‐ and late‐stage tumor that cannot be treated with conventional therapy [34]. Moreover, EGT has been demonstrated to be a novel immune vaccine features multiple immune activated components, including specific metals [35], electrotoxicity [36, 37] and gas signals [38, 39], which jointly reconstitute the microenvironment in immunosuppressive tumors. Specifically, EGT induces a massive ROS burst along with ICD effect excitation [40], releasing death‐associated molecular patterns (DAMPs) [41] and even inhibiting the accumulation of immunosuppressive myeloid‐derived suppressor cells (MDSCs) while increasing the proliferation of CD8^+^ T cells within the tumor [42, 43]. Inspired by these findings, ROS‐boosted therapies [44, 45] and gas‐constructed therapies [46, 47] have emerged as ICD‐amplified therapies [48]. There are sufficient reasons to infer that EGT is a well‐suited means of activating the immune response to effectively address the challenges posed by primary tumors, recurrence, and metastasis.

**SCHEME 1:**
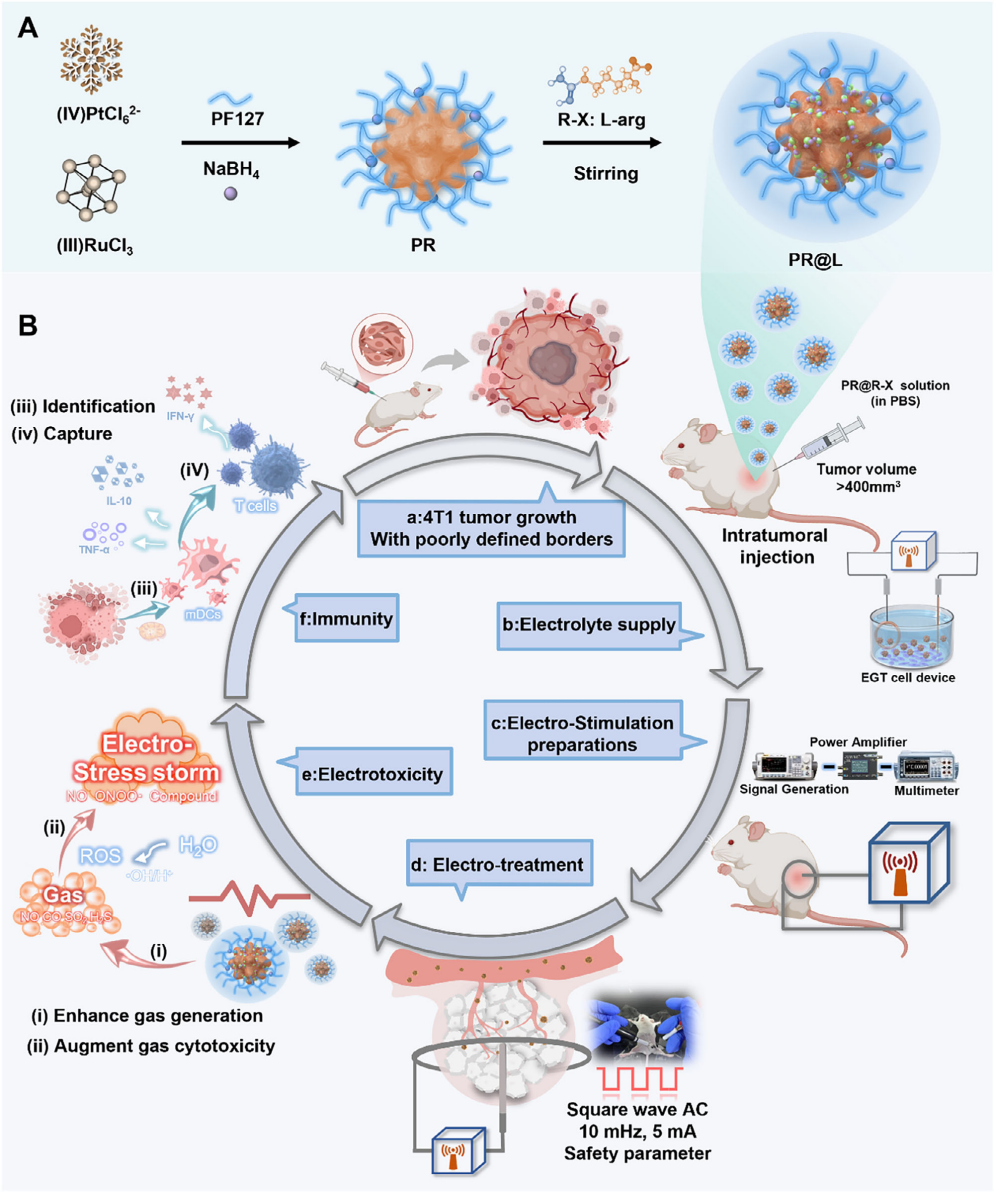
Schematic illustration of electric field‐induced cascade gas therapy platform. (A) Schematic illustration of the preparation of PR@L nanoparticle. (B) The workflow diagram illustrated a pioneering electric field‐induced cascade gas therapy that exerted electrotoxicity and immunity against large volume solid tumors via an electro‐stress storm. Tumor growth (a), electrolyte supply (b), electro‐stimulation preparations (c), electro‐treatment (d), electrotoxicity (e), and immunity function (f). R‐X for various gas donors (e.g., L‐arg, CORM‐3, L‐cysteine, and (NH_4_)_2_S_2_O_8_).

Herein, we proposed the concept of an electric field‐induced cascade gas therapeutic platform (EGT), which utilizes catalytic metal nanoparticles Pt‐Ru nano‐berries (PR) loading with multiple small molecular gas donors to combat large volume solid tumors through an electro‐stress storm (Scheme 1). The EGT therapeutic platform features an electric field‐responsive system that precisely controls the release of therapeutic gases and increases the gas yield, strengthening the effect of gas therapy. Furthermore, EGT‐based electrotoxicity augments the therapeutic potential of the released gases through forming toxicants with superior tumor specificity and efficiency, which in turn results in a significant electro‐stress storm to improve the performance of tumor treatments. Moreover, EGT has been demonstrated to be a novel adjuvant immunotherapy due to the capability of electro‐stress storm to activate the anti‐tumor immune responses, including identification and capture of electrotoxicity‐escaped tumor cells against tumor recurrence. Thus, this study demonstrates a pioneering approach applicable to diverse types of gas therapy, presenting a novel perspective for advancing the development of gas therapy.

## 2 Results and Discussion

### 2.1 Preparation and Morphological Characterization of Electrotoxic PR

**FIGURE 1:**
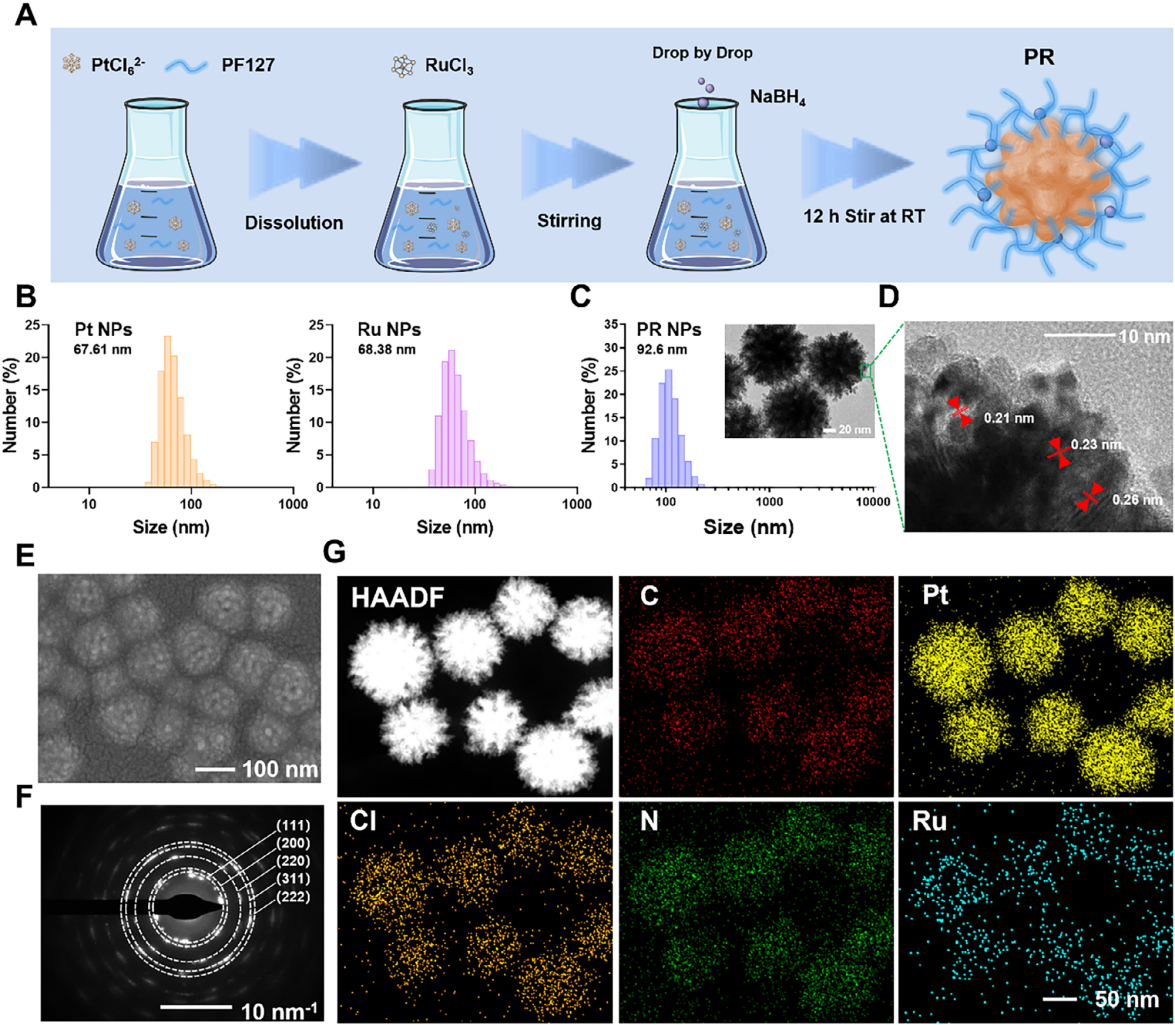
Preparation and morphological characterization of PR. (A) Schematic illustration of the construction of PR. (B) The DLS‐detected size data of Pt NPs and Ru NPs. (C) The DLS‐detected size data of and PR (inset: TEM image, scale bar: 20 nm). (D) A representative high‐resolution TEM image of PR in aqueous solution (scale bar: 10 nm). (E) The SEM (scale bar: 100 nm), (F) SAED (scale bar: 10 nm^−1^), and (G) STEM element mapping (scale bar: 50 nm) images of PR.

The optimization of electrotoxic Pt‐Ru nano‐berries (PR) was achieved through a one‐step oxidation‐reduction reaction, following the reported studies. In this reaction, metal ions were reduced by NaBH_4_ after the addition of a stabilizer polyethylene‐polypropylene glycol (PF127) at room temperature (Figure 1A) [49]. The well‐dispersed PR exhibited uniform and sphere‐branched structures with an average lateral dimension of 92.6 nm (Figure 1B,C). This resulted in a large specific surface area of PR nanoparticles, which facilitated the higher electrical conductivity and catalytic properties. High‐resolution transmission electron microscopy revealed highly crystallographic PR with lattice spacings ranging from 0.21 to 0.26 nm (Figure 1D). The PR were assembled from particles resembling spheres, as seen in the scanning electron microscope image (Figure 1E), hence named PR nano‐berries as their outlook like raspberries. The lattice pores of these PR nano‐berries also facilitated drug delivery, while their spherical structure with large specific surface area was conducive to improve the electrotoxic activity for the EGT platform. The selected‐area electron diffraction (SAED) pattern of the electrotoxic PR displayed some discrepancies with the PDF card of Pt (PDF#87‐0647) that commonly used in electrical therapy [50], which may be attributed to the incorporation of Ru into the crystal lattice (Figure 1F). Further proof of PR successful synthesis was confirmed by element mapping from scanning transmission electron microscopy (STEM), which showed uniform distribution and co‐location of Pt, Ru, and Cl in PR (Figure 1G).

### 2.2 Element, Valence‐Bond, Specific Surface Area, and Pore Analysis of PR

**FIGURE 2:**
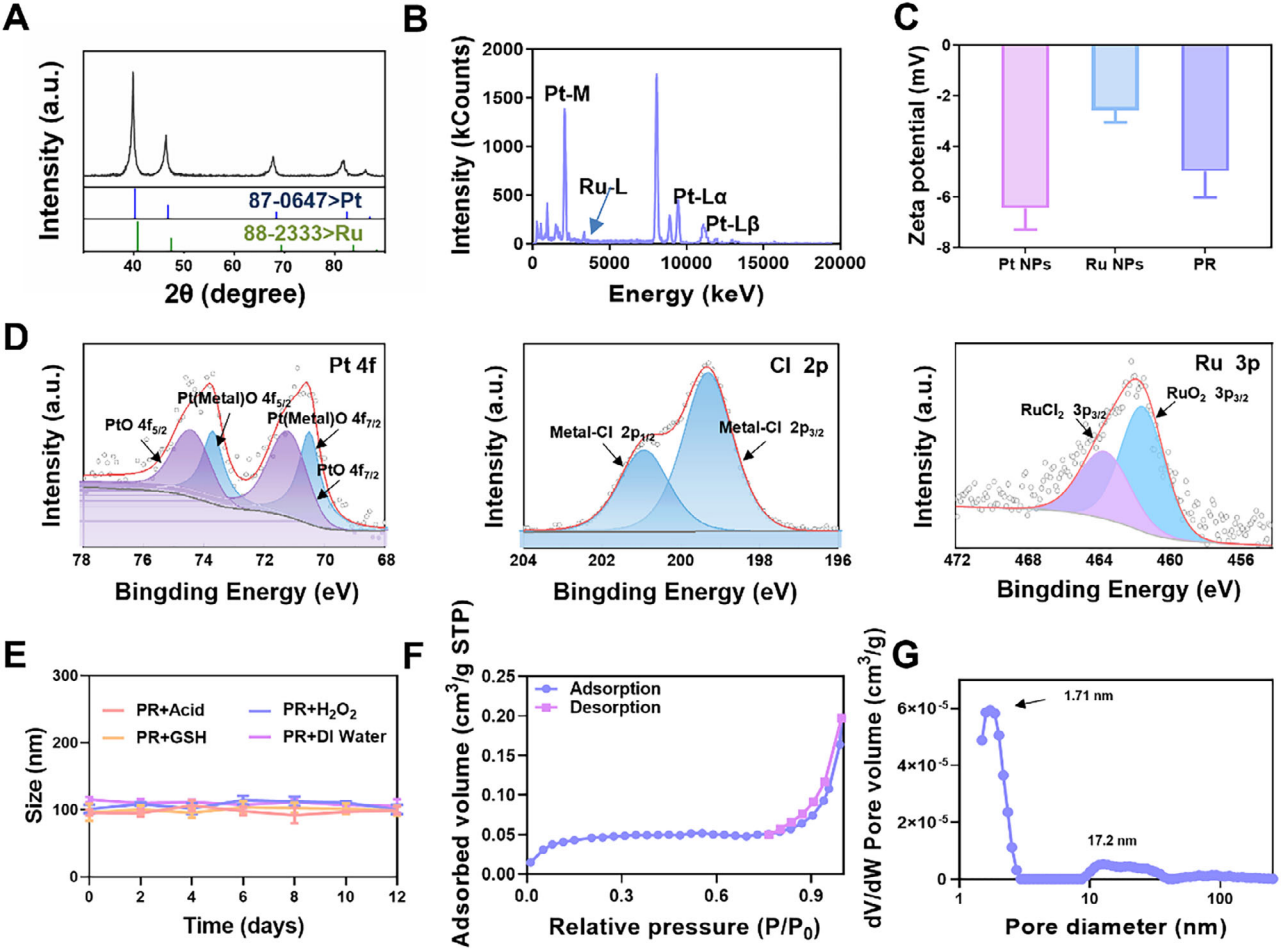
Element, valence‐bond, specific surface area and pore analysis of PR. (A) The XRD patterns and (B) X‐ray energy dispersive spectrum of PR. (C) Zeta potentials of Pt NPs, Pd NPs, and PR. (D) XPS data analysis of Pt (left), Cl (middle), and Ru (right) elements from PR. (E) The long‐term stability of PR monitoring by size distribution in different conditions. (F) The nitrogen adsorption/desorption curves and (G) the pore size distribution curves of PR.

Next, we performed a comprehensive analysis of the PR, including elemental composition, content, valence‐bond, specific surface area, and pore size distribution. As shown in Figure 2A, the X‐ray diffraction (XRD) pattern of PR aligned with the SAED pattern, supporting the possibility of PR forming solid solutions that maintained the original properties of these metals. Furthermore, the two additional peaks that were observed at 15°–30° are consistent with the PDF standard card (PDF#53‐1880) for poly (ethylene glycol), which is one of the components of PF127 (Figure S1). Thus, PF127 acts as both a structural guide and a colloidal stabilizer in the preparation of PR. The X‐ray energy dispersive spectrum of PR revealed the relative Pt and Ru contents (Figure 2B). The weak negative surface ζ‐potential of PR made them unable to move due to lack of driving force under the electric field (*E*), and also contributed to their stability in physiological/tumoral environment and minimized charge gradients during treatment (Figure 2C,E and Figure S2). X‐ray photoelectron spectroscopy (XPS) analysis data revealed the presence of metallic (0) and oxidation (+2) states for Pt, and oxidation (+4) states and chloride (+2) for Ru in PR (Figure 2D). As Ru NPs had no absorption at high wavelengths, the UV–vis absorption spectrum of PR was similar to that of Pt NPs at the same Pt concentration (Figure S3). According to the results of Brunauer–Emmett–Teller detection in Figure 2F, the PR exhibited a type IV adsorption isotherm, with an inflection point at the low‐pressure area (*P*/*P*_0_ = 0.9), indicating a strong interaction between the microporous surface of PR and nitrogen molecules. An H3‐type hysteresis band is observed with increasing relative pressure, suggesting an irregular pore structure stacked in the PR. An analysis of the pore size distribution revealed that the majority of the pores in the PR were micropores below 2 nm, with a relatively smaller number of mesopores located around 17.2 nm (Figure 2G). The microporous materials with large surface area and relatively high inside pressure facilitated the adsorption of the encapsulated drug, endowed PR nanoparticles that were dominated by microporous lattice stacks below 2 nm with excellent drug carrying potential.

### 2.3 Gas Generation and ROS Detection via Electrotoxicity on the EGT Platform

**FIGURE 3:**
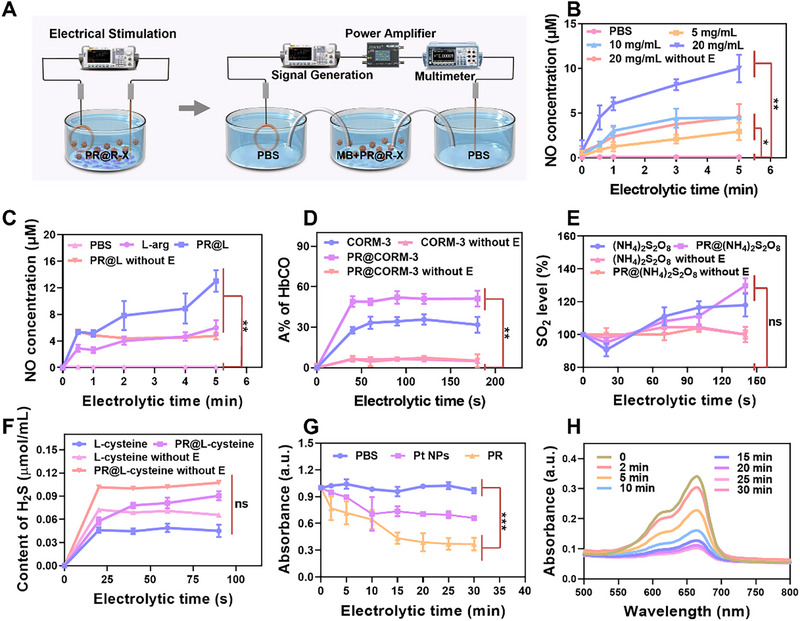
Gas generation and ROS detection via electrotoxicity on the EGT platform. (A) Schematic diagram of the device for electrocatalytic and electrolytic properties of EGT. (B) Different concentration L‐arg generation NO rates evaluated by the NO assay kit. (C) NO level in various formulations (PBS, L‐arg, PR@L‐arg) with and without electrical stimulation performance. Variety of gas donor molecules were produced with and without EGT performance to produce the corresponding (D) CO, (E) H_2_S, and (F) SO_2_. (G) EGT electrocatalytic properties of different formulations (PBS, Pt NPs, and PR groups) at different electrolytic time (H) detected by ROS catalytic degradation MB. *n* = 3 and *p < 0.05, **p < 0.01, and ***p < 0.001; ns: no significance.

As shown in Figure 3A and Figure S4, the advanced one‐piece EGT device was simpler and easier to operate than the previously used electrodynamic‐based device [51]. Briefly, the novel EGT platform was constructed to promote gas therapy through electrolysis‐induced electrotoxicity. Initially, L‐arginine (L‐arg) was applied in the EGT system as a routine gas donor to produce NO gas. Surprisingly, both electrical stimulation and increment concentrations of L‐arg resulted in a significant elevation of NO levels, with the NO production gradually stabilizing after a certain electrolysis time, providing a high expectation for the realization of gas generation and therapy in EGT performance (Figure 3B). Numerous studies have shown that the application of specific metals, such as Pt, Pd, and Ru, to catalysis via redox reactions can enhance the reaction rates, improving the main reactions that facilitate gas yield as well as reducing the side reactions [52, 53]. To further investigate the catalytic potential of PR on the EGT platform, L‐arg was loaded onto porous PR nano‐berries (PR@L) to examine the production rate and yield of NO. As expected, in the presence of EGT‐electrical stimulation, the rate, and yield of NO production in the PR@L group were almost twice as high as that in the L‐arg alone group. The gas yield of the PR@L without E group was essentially the same as that of the L‐arg group alone, but rates of NO gas production in the two groups were different at the outset, attributed to the good catalytic function of metal PR. The above results successfully demonstrate the feasibility of gas NO generation and increment in the EGT device (Figure 3C).

In order to develop an EGT platform with broader gas therapy applications, a variety of gas donors such as CORM‐3 (CO donor), L‐cysteine (H_2_S donor), and ammonium thiosulfate (SO_2_ donor) were applied to the device to investigate the production of gases with antitumor effects. Surprisingly, it was found that the gas donors loaded on PR groups (PR@CORM‐3, PR@(NH_4_)_2_S_2_O_8_) released more corresponding gases relative to PR@CORM‐3 without E or PR@(NH_4_)_2_S_2_O_8_ without E groups (Figure 3D,E). Although not significant in the detection of SO_2_, the application of EGT led to higher SO_2_ levels relative to (NH_4_)_2_S_2_O_8_ without E group and PR@(NH_4_)_2_S_2_O_8_ without E (Figure S5). Meanwhile, this was not consistent for all gas donors. In the exploration study of H_2_S, there was little difference in the H_2_S levels between the H_2_S donor alone group (L‐cysteine) and PR@L‐cysteine group with or without electrical stimulation (Figure 3F). L‐cysteine undergoes catalysis by cystathionine p synthase and cystathionine gamma cleavage enzyme in vivo, leading to the production of H_2_S gas. However, in vitro experiments failed to meet the required conditions, including the presence of both enzyme activities and related intermediate substrates. Obviously, the efficacy of EGT system is evidently influenced by multiple gas donors. Any gas donor that potentially decomposes under specific conditions can be deployed in the EGT system, such as through water solubilization, photo degradation, oxygen oxidation, and other mechanisms. In summary, the EGT platform enables the generation of multiple gases through the electrolysis of small molecular gas donors. The development of this EGT system has broadened the scope of potential gas therapy, and further research could lead to novel therapeutic approaches for tumor treatments.

On the other hand, the novel EGT platform made use of electrocatalysis to promote ROS generation for subsequent electrotoxicity. In order to investigate the activity of PR during the EGT process without the interference of electrolytic water, a double salt bridge electrocatalytic device system was constructed [54]. This detected system monitored the generation of ROS in the middle chamber by recording the UV–vis absorption spectra and the absorption intensity changes (at 664 nm) of the residual MB every 5 min during electrification. First, the electrotoxic property of Ru NPs alone was investigated, and it was observed that the MB absorption decreased with increasing electrocatalytic time, indicating that the metal Ru possessed catalytic capacity for EGT function (Figure S6). As seen in Figure 3G, the characteristic MB absorption intensity decreased only negligibly and slightly in the PBS group. Notably, PR significantly reduced the MB absorption compared to that of Pt group under electro‐stimulation with a 10 mHz square‐wave electric signal output, suggesting that there was a large amount of ROS production. At the same time, it demonstrated the promising electrocatalytic properties of PR for EGT function (Figure S7). Furthermore, the electrocatalytic properties‐based EGT of PR exhibited time‐ and current‐dependent characteristics (Figure 3H and Figure S8), confirming the necessity of electro‐stimulation conditions in EGT performance.

### 2.4 In Vitro EGT‐Based Electro‐Stress Storm on 4T1 Cells

**FIGURE 4:**
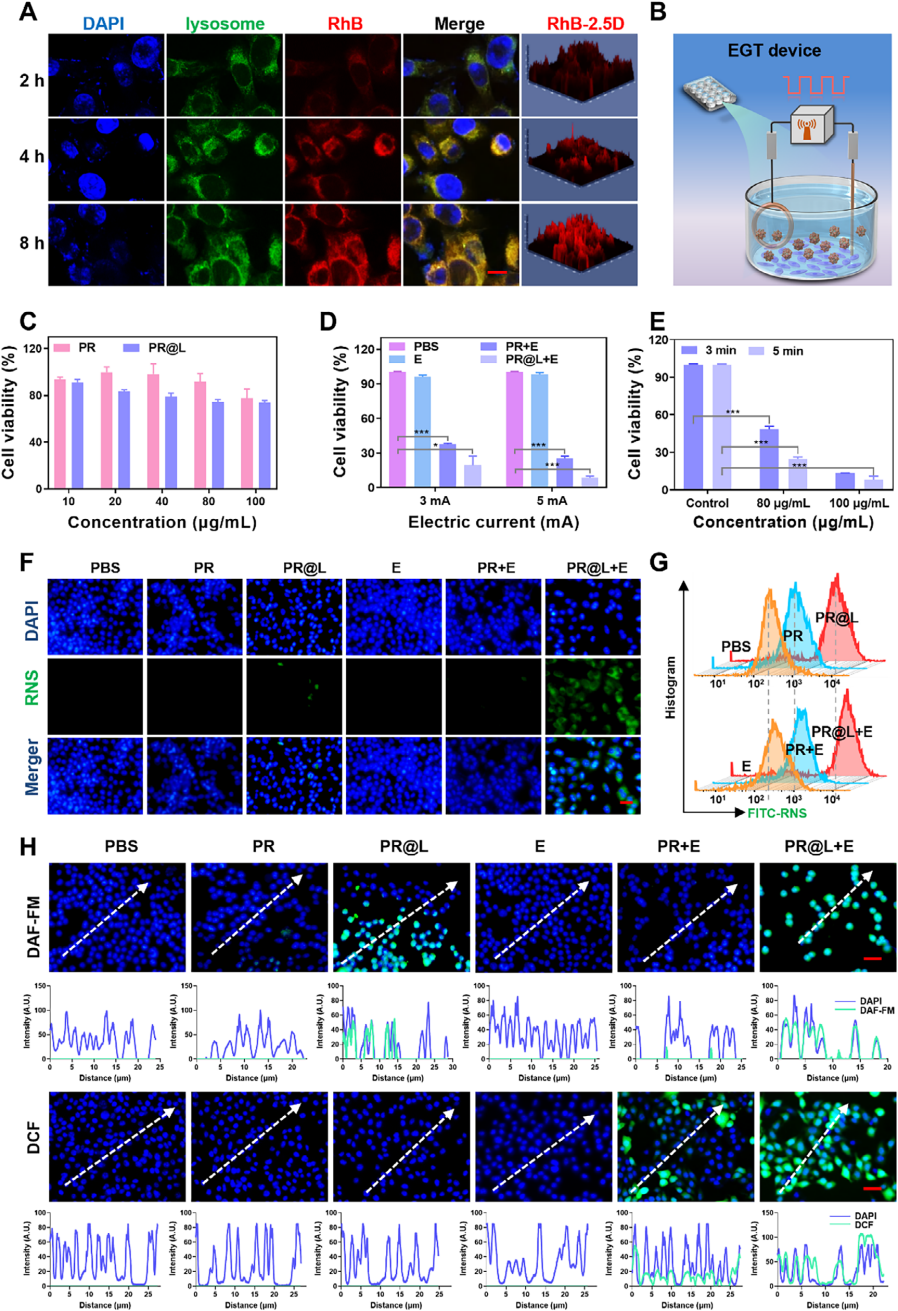
In vitro EGT performance and cytotoxicity of PR@L. (A) CLSM observation of RhB‐labeled PR internalized by 4T1 cells after different culture time. scale bar: 10 µm. (B) Schematic diagram of the experimental device for cytotoxicity evaluation of EGT system. (C) Cytotoxicity of 4T1 cells cultured with various formulations at diverse concentrations for 24 h. (D) Relative cytotoxicity of EGT‐treated 4T1 cells after incubation with different formulations (at 100 µg mL^−1^ Pt concentration) and (E) PR@L under various square‐wave AC parameters (10 mHz). (F) Intracellular RNS production after diverse treatments with or without electrical stimulation monitored by CLSM (scale bar: 50 µm) and (G) flow cytometer. (H) Intracellular ROS (DCF) and NO (DAF‐FM‐NO) CLSM images and corresponding fluorescence intensity profiles along the arrow (scale bar: 50 µm). *n* = 3 and *p < 0.05, **p < 0.01, and ***p < 0.001.

Subsequently, the electrotoxic PR nano‐berries were labeled with fluorescent probe rhodamine B (RhB) to monitor intracellular distribution. As shown in confocal laser scanning microscopy (CLSM) images, the RhB signals indicated for PR colocalized in lysosomes, suggesting the efficient cellular uptake of PR via the lysosome pathway (Figure 4A). Furthermore, a time‐dependent increase in cellular uptake behavior was observed, with intracellular internalization reaching its peak at 8 h. This observation was corroborated by flow cytometer and inductively coupled plasma mass spectrometry (ICP‐MS) analyses (Figure S9). An initial evaluation of material safety was conducted to ensure the biocompatibility of the PR. The results indicated no significant cytotoxicity on 4T1cells after 24 h‐treatment with PR, even at concentrations as high as 100 µg mL^−1^. However, the Ru NPs and PR@L displayed mild cytotoxicity, with cell viability reduced to 80% at high concentrations (Figure 4C and Figure S10). Furthermore, PR@L and PR@C exhibited comparable levels of toxicity (25%) at a concentration of 40 µg mL^−1^ (Figure S11). However, as the concentration of PR@C increased, a corresponding and significant increase in toxicity was observed. The results indicated that PR and PR@L showed negligible electrotoxic properties while good in vitro biocompatibility without electric field stimulation.

To assess the electrotoxic potential of the EGT platform to kill tumor cells under an electric field, we designed and established an experimental apparatus as depicted in Figure 4B. The cytotoxicity of 4T1 cells was evaluated after treatment with PR nano‐berries at relatively safe currents under various electric field parameters to investigate the electro‐stimulated EGT potential in vitro. As expected from Figure 4D, PR was effective in killing 4T1 tumor cells when stimulated with electric fields. Inspiringly, PR@L, a metal nanoparticle modified with L‐arginine, presented an enhanced ability to kill tumor cells compared to PR alone, which may be ascribed to the adjuvant‐enhanced killing effect of NO produced by L‐arginine [55]. Additionally, the study demonstrated that the cytotoxicity of PR@L could be further enhanced by increasing the metal concentration, electro‐stimulated time, and square wave AC intensity, verifying the adjustable electrotoxic activity of PR@L (Figure 4E). These findings suggested that PR@L has potential as an electro‐triggered nano‐biomaterials for tumor EGT treatment, and that its electro‐stimulated effect can be fine‐tuned to enhance its efficacy. In summary, the initial success of the EGT platforms at the cellular level was achieved.

Then, to observe the electrical stress storm, fluorescence probes were used to investigate relevant indicators of electro‐stress storm, such as RNS (ONOO·), NO, and ROS. The BBoxiProbe O52 probe was employed to detect the intracellular RNS content and understand the killing mechanism of the EGT effect. The CLSM observation and flow cytometer results showed that there were almost no RNS fluorescence signals generated in the PR and PR@L groups, indicating the safety of these catalytic metal PR nanomaterials without electro‐stimulation (Figure 4F,G). However, weak RNS levels were detected in the PR@L group due to the spontaneous NO production from L‐arg in the tumor oxidative environment. A considerable amount of green fluorescence was observed in the PR@L+E group, indicating substantial RNS production, which suggested that both NO and ROS are produced, reacting to form highly potent RNS in the PR@L+E group under electric field stimulation. Meanwhile, DCFH‐DA and DAF‐FM DA probes were used to detect ROS and NO levels in the cells, respectively. Consistent with the RNS results, the cells exhibited the ability to produce both ROS and NO upon receiving nano‐berries and electrical stimulation (PR@L+E), confirming the presence of RNS from NO and ROS, demonstrating the electrotoxicity of PR@L in the novel EGT therapeutic platform (Figure 4H, Figures S12 and S13). These findings provided insight into the cytotoxic mechanism of EGT, whereby tumor killing effect was achieved through a significant electro‐stress storm, which enhanced the efficiency of gas generation and increased cytotoxicity. This highlighted the promising application potential of PR@L+E as a responsive antitumor platform.

Additionally, an annexin V‐FITC/propidium iodide (PI) double staining assay was conducted to study the apoptosis and necrosis effect of EGT‐based electro‐stress storm. The fraction of living cells in the PBS, E, PR, and PR@L groups was high, consistent with the MTT assay results. However, the percentage of apoptotic and necrosis cells in the PR@L group was significantly higher (66.8%) than that of PR+E group (50.16%) (Figure S14). These findings once again demonstrated that the augmented electrotoxicity effect of RNS produced by PR@L under electric field stimulation was capable of inducing apoptosis and necrosis, leading to the death of tumor cells.

### 2.5 In Vivo EGT Performance on Tumor‐Bearing Mice

**FIGURE 5:**
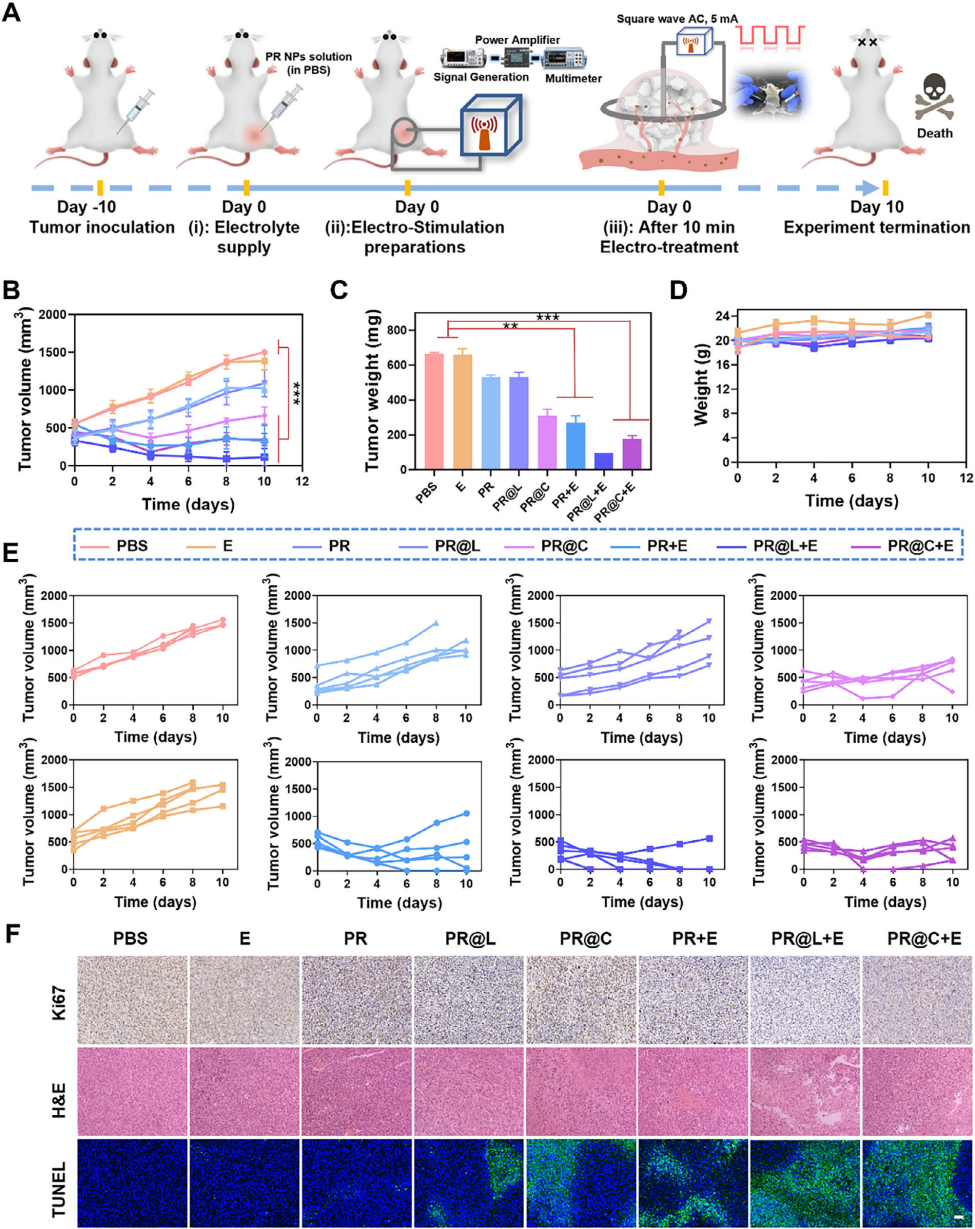
In vivo EGT treatment for solid tumor with large size by electro‐stimulating gas donor‐encapsulated PR. (A) Schematic illustration of in vivo EGT treatment operation. (B) Tumor growth curves of 4T1 tumor‐bearing mice after different treatments (*n* = 5). (C) Average tumor weights and (D) mouse weight at the end of the experiment (at day 10, *n* = 5). (E) Tumor growth curves of individual mouse from each group. (F) Ki67, H&E, and TUNEL staining analyses of tumor tissue slices collected from different groups after treatment. Scale bar: 50 µm. *p < 0.05, **p < 0.01, and ***p < 0.001.

Based on the promising results of internalization, cytotoxicity, and biocompatibility in vitro, we conducted further investigations to assess the antitumor performance of gas donor‐loaded PR as a platform for EGT under an electric field. Numerous research studies have demonstrated that the achievements of gas therapy in the field of tumor treatment, for instance, NO can reduce the tumor viability by inducing intracellular nitrosative stress [56], H_2_S is able to strengthen the intracellular inflammatory response by specifically inhibiting peroxidase activity [57], and CO is capable of destroying tumor cells by inhibiting cytochrome c oxidase and disrupting protein synthesis [58]. To this end, we utilized PR nano‐berries to load commercial NO (L‐arginine) and CO (CORM‐3) gas donors, respectively, as representative electrocatalytic nanomedicines to investigate the electrical stimulation‐induced EGT effects on tumor‐bearing mice, with the aim of developing a universal gas donor‐carrying platform for gas combination therapy. A group of 40 mice bearing subcutaneous 4T1 tumors with an average initial size of approximately 400 mm^3^, which was considered a large tumor size that does not respond well to most conventional treatment options [59], were randomly divided into eight groups (*n* = 5). After intertumoral injection of different nano‐formulations, the sole EGT treatment was administrated and recorded as day 0. The treatment apparatus was shown in Figure 5A and involved placing a ring electrode around the tumor and inserting another electrode into the center of the tumor. The electrodes were then connected to the equipment for EGT effect. Tumor growth curves and average mice weights were monitored throughout the treatment. Mice were humanely euthanized when average tumor volume in the control (PBS) group reached a volume of about 1500 mm^3^ and the experiments were terminated. The experiments were strictly performed under the guidelines evaluated and approved by the Animal Ethics Committee of Southern Medical University, P. R. China.

As shown in Figure 5B,E, it was found that the PR@L+E group exhibited more effective tumor growth suppression compared to the PR+E group. And all of the tumors treated by PR@L+E almost completely disappeared from the data on average tumor weight (Figure 5C), indicating that the PR@L‐based EGT platform exerted efficacious electro‐stress storm function for anti‐tumor. Similarly, an excellent antitumor activity was observed in the PR@C+E group, which was ascribed to the synergistic effect of CO and ROS through oxidation coordination. This process entails the activation of oxygen‐containing gas molecules by ROS, resulting in their subsequent coordination reactions with specialty metals. Additionally, both PR@L+E and PR@C+E groups showed smaller tumor size and tumor weight compared to other groups (Figure S15). No difference was observed in the tumor volume of 4T1 tumor‐bearing mice from the PBS, PR, and PR@L groups, indicating the high biosafety of the PR‐based nano‐berries in the absence of an electric field, which was consistent with the cytotoxicity results. However, the PR@C group also displayed promising tumor growth inhibition, potentially attributable to the antitumor effect of individual CO gas from CORM‐3 [60]. Furthermore, in the group where only the electronic field was applied (E group), there was no significant difference in tumor volume compared to the PBS group, implying that electrical stimulation alone would not affect tumor growth or damage the health of mice.

It was observed that there were no significant variations in body mass or survival rates among the PR@R‐X+E groups, which confirmed the good safety performance of PR nanoparticles as drug carrier platform in EGT system to a certain extent (Figure 5D and Figure S16). And then, H&E, TUNEL staining and immunohistochemistry Ki67 staining were performed on tumor slices to investigate the therapeutic effects of various treatments. As expected, both the PR@L+E and PR@C+E groups exhibited more efficient therapeutic effects compared to the other formulations. These results were supported the lower percentage of positive cells in Ki67 staining slices (brown nuclei represent Ki67‐positive cells), the increased dead cell cavities or massive reduced cell nucleus in H&E staining tissues, and the stronger green fluorescence apoptotic markers and more nuclei fragments in the TUNEL stained images (Figure 5F). The above results demonstrated that PR@L or PR@C possess a powerful tumor cell killing capability depended on EGT‐based electro‐stress storm effect, providing strong evidence for electro‐gas synergistic tumor therapeutic effects.

### 2.6 EGT‐Induced In Vivo Immunogenic Effect

**FIGURE 6:**
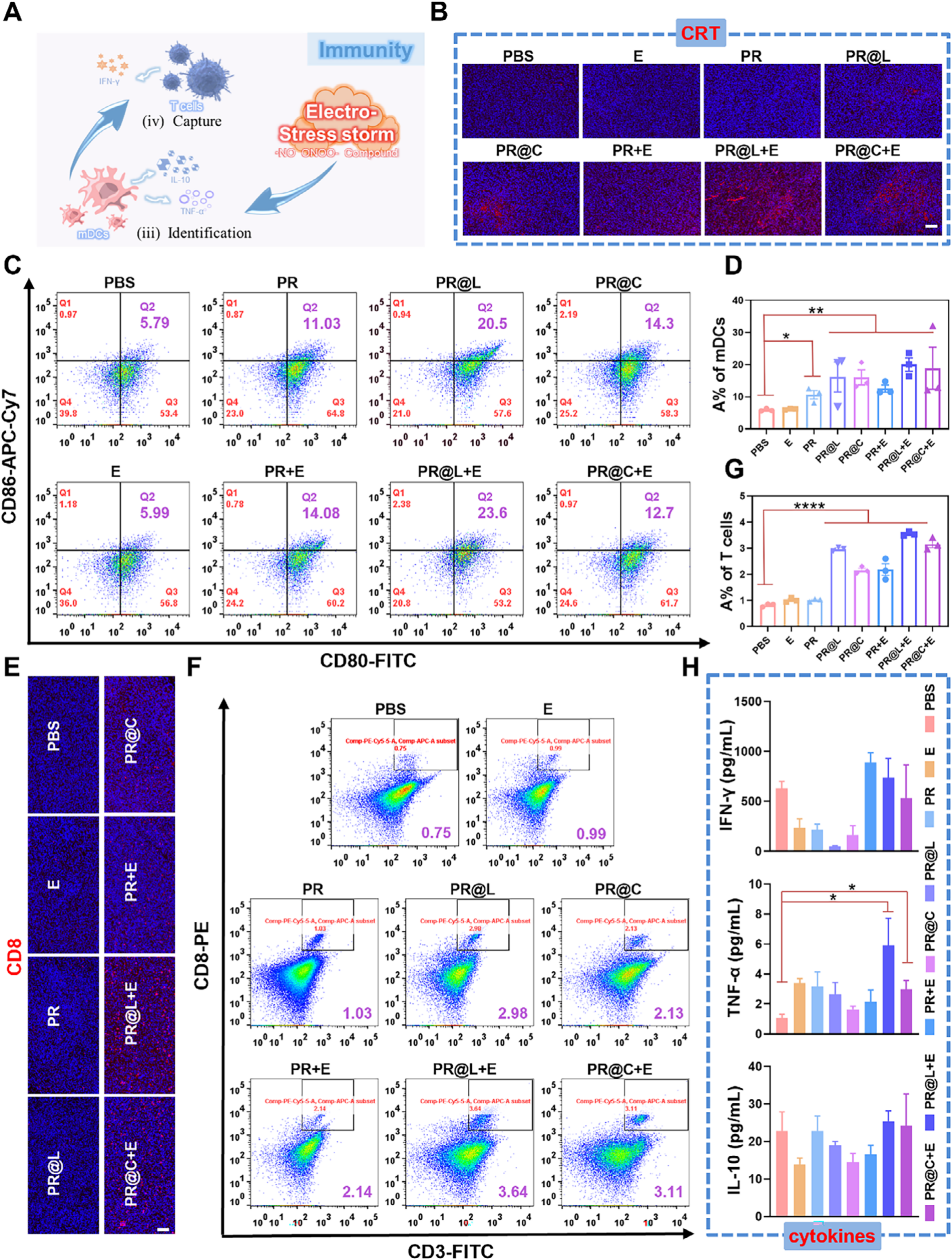
EGT‐induced in vivo immunogenic effect boosted tumor immunotherapy. (A) Scheme illustration of immune activation mechanisms of the EGT platform through releasing immunogenic DAMPs and generating PR@L‐mediated electro‐stress storm. (B) Immunofluorescence analysis for in situ CRT exposure in 4T1 tumors from mice after various treatments. Scale bar: 100 µm. (C) Flow cytometric analysis and (D) corresponding quantitative analysis (*n* = 3) of DCs maturation (gated on CD11c^+^CD80^+^CD86^+^) in tumor draining lymph node cells collected from mice 10 days after treatments. (E) Immunofluorescence analysis (scale bar: 100 µm), (F) flow cytometric analysis, and (G) corresponding quantitative analysis (*n* = 3) of infiltrating CD8^+^ T cells (gated on CD45^+^CD3^+^CD8^+^) in tumor tissues from mice after various treatments. (H) ELISA analysis of cytokines in serum from 4T1 tumor‐bearing mice, including TNF‐α, INF‐γ, and IL‐10 (*n* = 3). *p < 0.05, **p < 0.01, and ***p < 0.001.

In this EGT platform, PR@R‐X can significantly enhance gas generation efficiency and cytotoxicity at the tumor site, ultimately destroying the tumor cells by electro‐stress storm. Meanwhile, electro‐stress storm effect has been proven to boost the immunogenicity of tumor cells via a specific cell death modality, including release of immunogenic damage‐associated molecular patterns (DAMPs), which further promoting maturation of DCs and recruitment of cytotoxic CD8^+^ T cells. These mechanisms enhance the recognition and capture of tumor cells that escape from electrotoxicity. Concurrently, the immune cells secrete various cytokines, such as tumor necrosis factor‐α (TNF‐α), interleukin‐10 (IL‐10), and interferon‐gamma (IFN‐γ), which potentiate immunotherapy through a positive feedback loop (Figure 6A).

Immunofluorescence staining of 4T1 cell and tumor slices were performed to investigate the role of immunogenic ICD effect which was represented by characteristic DAMPs (e.g., CRT and HMGB1) during EGT‐based electro‐stress storm strategy. The results showed a slight increase in CRT immunofluorescence signal in the PR@L and PR@C groups, representing a small amount of CRT exposure compared to the control group (Figure 6B and Figure S17). Meanwhile, when electrical stimulation was applied, a large amount of CRT was ectopically turned on the cell membrane in the PR@L+E and PR@C+E groups relative to the PR+E group, demonstrating an activation of the DAMPs‐based immune response resulting from the electro‐stress storm effect of gas therapy. Additionally, HMGB1 was predominantly located in the nucleus in non‐treated cells. However, significant translocation of HMGB1 from the nucleus to the extracellular environment was observed in PR@L+E and PR@C+E groups (Figure S18). These findings provided evidence that electrotoxicity effect of electric field‐induced cascade gas therapy produced by the versatile EGT platform may boost the immunogenicity of tumor cells by synergistically increasing the expression of DAMPs.

To ensure the rapid identification of electrotoxicity‐escaped tumor cells, the effect of EGT‐induced electro‐stress storm on the maturation of DCs was examined. Flow cytometry was performed on a single cell suspension of tumor tissue to study the marker expression of DCs. The results showed that the maturation rate of DCs increased ≈2–5 fold in all nanoparticle groups (PR, PR@L, and PR@C groups) as well as in the groups receiving electrical stimulation (PR+E, PR@L+E, and PR@C+E groups) compared to the PBS group (Figure 6C,D). The increase in maturation rate was only slightly higher in the electrostimulation group than in the electro‐absent group, reaching up to 23.6% (PR@L+E group). The above data proved that PR@R‐X+E in the EGT system can activate DCs maturation to achieve identification, providing a solid foundation for the subsequent elicitation of systemic immunotherapy. Furthermore, an outstanding immunity not only activates DCs, but also plays an important role in promoting the intertumoral infiltration of CD8^+^ T cells, as reported in a wide range of studies. Thus, flow cytometry and immunofluorescence staining were performed to study the infiltration population and protein expression during the immune activation, respectively. Significant improvement of CD8^+^ T cell infiltration (purple fluorescence enhancement and T cell numbers increase) was observed in tumor tissues of mice receiving EGT treatment (PR@L+E and PR@C+E groups) (Figure 6E,F), fully demonstrating that the electro‐stress storm effect of EGT platform could evoke a strong protective anti‐tumor T cell immunity to complete the final capture work (Figure 6G). The above results showed that the EGT‐based electro‐stress storm not only attacks large volume solid tumors but also triggers an immune response against tumor recurrence.

In addition, the secretion of cytokines plays an essential role in the activation and progression of the immune responses. To detect the activation of systemic immunity, the levels of inflammatory cytokines in serum, including IFN‐γ, TNF‐α, and IL‐10, were measured. It has been demonstrated that both IFN‐γ and TNF‐α can promote the activation and tumor tropism of T/NK cells, whereas IL‐10 can suppress antitumor immunity via direct inhibition of T‐cell functions and indirect expansion of immunosuppressive Tregs and MDSCs. As illustrated in Figure 6H, the increased IFN‐γ levels were observed in the PR+E, PR@L+E, and PR@C+E groups. Meanwhile, a significant increase in the TNF‐α secretion was noted in the PR@L+E and PR@C+E groups. This result suggested that the electric field‐induced cascade gas therapy platform was effective in stimulating inflammation and increasing tumor immunogenicity. Conversely, the IL‐10 levels remained unaltered, likely due to the sampling and testing at the end of the treatment cycle. These results demonstrated that PR@R‐X‐based EGT systems were able to effectively trigger an immune response against tumor growth.

### 2.7 In Vivo Pathological and Biological Safety Study for the EGT Treatment

To further evaluate the long‐term biological safety of PR@L‐based EGT performance in vivo, major organs and serum were collected for H&E staining and serum biochemistry tests, respectively. Alanine aminotransferase (ALT) and aspartate aminotransferase (AST) were used to assess liver injury, while creatinine (CREA) and urea (UREA) were utilized to assess renal function. Creatine Kinase (CK) was able to indicate damage to skeletal muscle or cardiac muscle. The results showed no pathological changes in major organs of mice receiving different treatments (E, PR, PR@L, PR@C, PR+E, PR@L+E, PR@C+E) (Figure S19). Serum biochemical markers of mice in each group were within the normal range, although there were slight fluctuations. No significant differences were found when compared with the PBS group (Figure S20). Overall, the above results fully demonstrated the low toxicity of the PR‐based materials and established a solid foundation for the subsequent clinical safety evaluation of EGT.

## 3 Conclusions

We have developed a brand‐new EGT platform in this study, which was capable of exploiting advanced metal nano‐berries (PR@L) to exert electrotoxicity and immune activation via tumor electro‐stress storm for efficient tumor therapy. L‐arg and CORM‐3, as representative gas donors, were first applied to the EGT platform in combination with electrical field stimulation. On the one hand, the EGT platform was triggered to release the therapeutic gas components from the gas donors in response to the adjustable electrical stimulation, allowing for flexible spatiotemporal release and yield of the therapeutic gases. On the other hand, these therapeutic gas components in PR nanoberries‐mediated EGT can superimpose electrotoxicity via forming oxidation coordination (CO and ROS) or generating new therapeutic components (RNS reacted from ROS and NO) to ablate large volume tumors based on EGT therapy. More excitingly, the stimulated electro‐stress storm further provoked the immune vaccine effect of the EGT platform against non‐solid tumors or residual tumors. Therefore, this study presented a promising joint platform with broad applications in electro‐nanotechnology and gas co‐therapy, opening up new avenues for further research.

## 4 Materials and Methods

### 4.1 Materials

Methylene blue (MB), chloroplatinic acid hexahydrate (H_2_PtCl_6_·6H_2_O), rhodium (III) chloride hydrate (RuCl_3_·3H_2_O), polyethylene‐polypropylene glycol (PF127), sodium borohydride (NaBH_4_), CORM‐3(≥98%), rhodamine B, potassium chloride (KCl, 99.8%), 3‐(4,5‐dimethylthiazol‐2‐yl)‐2,5‐diphenyltetrazolium bromide (MTT, 97%) were acquired from Aladdin. 2’‐(4‐Ethoxyphenyl)‐5‐(4‐methyl‐1‐piperazinyl)‐ 2,5’‐bi‐1H‐benzimidazole trihydrochloride bisBenzimide (Hoechst 33342, 98%) and RNS test kit were purchased from Beijing Solarbio Science & Technology Co., Ltd. L‐arginine was purchased from Macklin Industrial Co., Ltd., sodium hydrosulfide hydrate (NaHS·*x*H_2_O) from Shanghai Acmec Biochemical Co., Ltd., sulfur dioxide quick test kit, carbon monoxide hemoglobin test kit (colorimetric method) and micro hydrogen sulfide kit available from Guangzhou Zero One Biological Technology Co., Ltd. Fetal bovine serum was provided by Inner Mongolia Opcel Biotechnology Co., Ltd. Cell culture dishes, RPMI‐1640 medium and trypsin were purchased from Viva Cell (Shanghai, China). Cell Counting Kit‐8 was provided by Sevenbio (Beijing, China). Annexin V‐FITC/Propidium iodide (PI) apoptosis detection kit, NO detection kit, and DCFH‐DA were obtained from Beyotime Biotechnology. Calcein‐AM/PI double stain kit and lysotracker green DND‐26 were obtained from YEASEN Biotechnology. The ELISA kits of IFN‐γ, TNF‐α and IL‐10 were obtained from Jiangsu Jingmei Biological Technology Co., Ltd. Anti‐CD8 alpha antibody was purchased from Abcam. Goat anti‐mouse IgG and Goat anti‐rabbit IgG were purchased from Biosynthesis Biotechnology Inc. (Beijing, China). Other antibodies were purchased from eBioscience and Biolegend. All antibodies used for Western blot were purchased from Abcam (UK).

### 4.2 Cell Lines and Animal

4T1 mouse breast cancer cell line was provided by the American Type Culture Collection. The 4T1 cells were cultured in RPMI 1640 or DMEM (high glucose), supplemented with 10% FBS and pen/strep. Cells were cultured in a constant temperature incubator with 5.0% CO_2_ at 37°C.

Balb/C female mice (6–8 weeks old) weighing between 20–25 g were purchased from the Animal Experimentation Center of Southern Medical University, Guangzhou, China. All animal experiments were performed under the guidelines evaluated and approved by the Animal Ethics Committee of Southern Medical University, P. R. China (Approval Number: KYKT2021‐015).

### 4.3 Preparation of Pt NPs or Ru NPs and PR Nano‐Berries (PR)

The reactions were synthesized following a one‐pot method. There is a slight difference at the first step. Briefly, 75 mg H_2_PtCl_6_·6H_2_O or 30 mg RuCl_3_·3H_2_O and 64 mg PF127 in a conical flask were dissolved in 5 mL DI water. Then, 22 mg NaBH_4_ pre‐dissolved in 36 mL ice‐cold water was dropwise added into the mixture on the magnetic stirrer and continuously reacted for 12 h at room temperature. Finally, the PR were collected by an ultrafiltration centrifugation device (MWCO: 100 kDa) and washed three times with DI water and ethanol (1:1) to remove the residuals.

PR conjugated to rhodamine B (RhB@ PR) probes were prepared for cellular uptake in vitro. 5 mL of PR stock solution and 1 mg of RhB probe were stirred overnight. A simple method was employed to prepare gas donors loaded on PR (PR@R‐X). 1 mL PR concentrated stock solution (Pt: 20 mg mL^−1^) and different gas donors (5 mg L‐arg, L‐cysteine, and (NH_4_)_2_S_2_O_8_ or 2 mg CORM‐3) were mixed overnight. Then the mixture was put into a dialysis bag (MWCO: 3500 Da) to remove the excess reagent.

### 4.4 Electro‐Catalytic Activity Studies

The degradation rates of methylene blue (MB) were used to detect the electro‐catalytic properties of PR with a function signal generator. Shortly, different amounts of PR were added to 2 mL PBS containing 30 µM MB in the middle well. The function signal generator, the multimeter, and Pt electrodes were connected in turn. The salt bridge and the PBS buffer were employed in vitro experiments to provide an electrolyte. 10 mHz, a square‐wave electrical signal with a different voltage was applied. After powering on for a different period, 30 µL of the mixture was collected and diluted with PBS to 330 µL. The absorbance spectra of MB were characterized by UV–vis spectroscopy.

### 4.5 Electro‐Gas Property Investigation

There are some slight differences from electro‐catalytic activity studies; one end of the device uses Pt sheet electrodes in an electro‐gas property investigation. All reaction reagents are placed in the same chamber, including gas donor reagents, PR, or condition‐specific PBS buffer solution (e.g., H_2_O_2_). 10 mHz, a square‐wave electrical signal with a different voltage was applied. 100 µL of the mixture was collected and detected with various gas detection kits at different electrolysis time points. NO donor L‐arg, CO donor CORM‐3, SO_2_ donor (NH_4_)_2_S_2_O_8_, H_2_S donor NaHS·*x*H_2_O.

### 4.6 In Vitro Studies

#### 4.6.1 Cell Internalization

To evaluate PR intracellular uptake in vitro, we used CLSM, flow cytometry, and ICP‐MS. 4T1 breast tumor cells were exposed to RhB‐loaded PR for different durations (2, 4, and 8 h) and then rinsed thrice with PBS. CLSM was performed after 4T1 breast tumor cells were stained with lysosome‐FITC and DAPI. Cells were directly uploaded to the flow cytometer after resuspension into a single‐cell suspension with PBS. ICP‐MS was conducted after 4T1 breast tumor cells were oxidized and decomposed by fuming nitric acid and hydrogen peroxide.

#### 4.6.2 Cell Viability Assays

MTT assays were used to evaluate cytotoxicity in vitro. Simply, 4T1 breast tumor cells were seeded onto 96‐well plates at 5000 cell/well and treated with different various formulations (PR, PR@L) at different concentrations (Pt: 10, 20, 40, 80, and 100 µg mL^−1^; Ru: 4, 8, 16, 32, and 40 µg mL^−1^) for 24 h. 10 µL of 5 mg mL^−1^ MTT were then added into each well and incubated for 4 h. 150 µL DMSO was added into each well to dissolve the resulting formazan and absorbance read at 490/570 nm using a microplate reader.

For EGT treatment, 4T1 cells were plated in 24‐well plates and incubated with different formulations, including PBS, E, PR+E, PR@L+E (Pt: 100 µg mL^−1^). After 4 h, 4T1 cells were treated with EGT plan under direct a square wave AC electric field (10 mHz, 100 s) 3 min and indirect a square wave AC electric field (10 mHz, 100 s) at 2 or 5 mA for 3 or 5 min.

#### 4.6.3 Intracellular NO Level Generated by EGT Treatment

DAF‐FM‐DA is a NO fluorescent probe, which can be broken down into DAF‐FM by intracellular esterase. DAF‐FM can react rapidly with NO to produce green fluorescence benzotriazole derivative. 4T1 breast tumor cells on 24‐well plates were treated with different formulations (PBS, E, PR, PR+E, PR@L, PR@L+E). After 4 h, 4T1 cells were treated with EGT therapy under a square wave AC electric field (10 mHz, 100 s) at 5 mA for 5 min. Subsequently, 5 µM DAF‐FM‐DA probe was added for 20 min at 37°C. Finally, cells were instantly stained with DAPI for CLSM.

#### 4.6.4 Detection of EGT‐Based Reactive Nitrogen (RNS) In Vitro

The RNS assay kit was used to assess the RNS production. RNS levels were quantified by CLSM and flow cytometry. All experimental operations are similar to EGT treatment, but there was a little difference. 4T1 breast tumor cells on 24‐well plates were treated with different formulations (PBS, E, PR, PR+E, PR@L, PR@L+E). BBoxiProbe O52 probe was added 30 min before treatment. After 4 h, 4T1 cells were treated with EGT therapy under a square wave AC electric field (10 mHz, 100 s) at 5 mA for 5 min. Subsequently, cells were instantly stained with DAPI for CLSM.

### 4.7 In Vitro Studies on Tumor Cells

All operations are the same as above. There were a few differences. 4T1 breast tumor cells on 24‐well plates were treated with different formulations (PBS, E, PR, PR+E, PR@L, PR@L+E). After 4 h, 4T1 cells were treated with EGT therapy under a square wave AC electric field (10 mHz, 100 s) at 5 mA for 5 min. Annexin V‐FITC/PI apoptosis detection kit was used to evaluate the ability to kill tumor cells.

### 4.8 In Vivo Electro‐Gas Therapy

6/8‐weeks‐old female Balb/C mice were subcutaneously inoculated with 100 µL PBS of 4T1 cells. When the tumor volume reached ≈400 mm^3^, 4T1 breast tumor cells tumor‐bearing Balb/C mice were divided into eight groups, and intratumorally treated with PBS, E, PR, PR+E, PR@L, PR@L+E, PR@C, and PR@C+E (Pt at 2 mg kg^−1^, L‐arg at 12.5 mg kg^−1^, CORM‐3 at 5 mg kg^−1^, 50 µL). The mice were thoroughly anaesthetized, and the fixator limbs were immobilized prior to treatment. Subsequently, the device was connected with a ring‐shaped platinum electrode attached to the exterior of the tumor and a platinum needle electrode inserted into the interior of the tumor at a depth of 8–10 mm. The PBS solution was utilized as a solvent to dissolute of nano‐berries to provide electrolyte in vivo experiments. After 10 min, these mice suffered EGT treatment. In this experiment, body weight and tumor volume were recorded and monitored for 10 days. Tumor volume was calculated using the equation: (tumor length) × (tumor width)^2^/2. Animals were euthanized once the volume of a tumor reached 1500 mm^3^. Tumors were harvested for H&E and immunohistochemical staining operated by Servicebio Biological Technology, ELISA assay operated by Jiangsu Jingmei Biotechnology Co., Ltd., Yancheng, China.

### 4.9 Ex Vivo Flow Cytometry Analysis of Immune‐Related Markers

At 10 days after treatment in each group, tumor slices were collected and homogenized within PBS (pH 7.4) to obtain single‐cell suspensions after treatment. Cells were first stained with live/dead staining, followed by the corresponding antibodies of anti‐CD3‐FITC, anti‐CD45‐APC, anti‐CD4‐Cy7, and anti‐CD8‐PE incubation. Subsequently, single‐cell suspensions were analyzed on a flow cytometer.

### 4.10 Statistical Analysis

All data results are expressed as mean ± standard deviation. Comparisons between groups were performed using one‐way analysis of variance (ANOVA), with *p* < 0.05 considered statistically significant.

## Author Contributions

Gui Chen and Wenjia Zhang contributed equally to this work. Gui Chen and Meng Yu conceived and designed the project. Gui Chen, Wenjia Zhang, Manchun Wang, Fengling Zhang, and Mengliang Zhu processed the experiments and collected the data. Gui Chen, Yan Tang, Yixian Xie, and Wen Ma analyzed the data and drafted the manuscript. Peter Timashev conducted the supplementary experiment and analyzed the data. Massimo Bottini and Yingqiu Xie embellished the article. Xing‐Jie Liang, Meng Yu, and Zhiqiang Yu revised the manuscript.

## Conflicts of Interest

The authors declare no conflicts of interest. Xing‐Jie Liang is a member of the *Exploration* editorial board, and he was not involved in the handling or peer review process of this manuscript.

## Supporting information




**Supporting File 1**: exp270104‐sup‐0001‐SuppMat.docx.

## Data Availability

The data that support the findings of this study are available from the corresponding author upon reasonable request.
