# Supplementary material for: A Groundbreaking Electric Field‐Induced Cascade Gas Therapy Against Large Volume Solid Tumor Through Electro‐Stress Storm
Source: Exploration (Beijing). 2025 Dec 21;5(6):20240410. doi: 10.1002/EXP.20240410 (PMC12752579; doi:10.1002/EXP.20240410)
Supplement: Supplementary file 1 — Supporting File 1: exp270104‐sup‐0001‐SuppMat.docx. [file EXP2-5-20240410-s001.docx]

**A Groundbreaking Electric Field-induced Cascade Gas Therapy Against Large Volume Solid Tumor Through Electro-stress Storm**

*Gui Chen^1^, Wenjia Zhang^2^, Manchun Wang^3^, Fengling Zhang^3^, Mengliang Zhu^4,5^, Yan Tang^1^, Yixian Xie^3^, Wen Ma**^1^, Peter Timashev^6^, Massimo Bottini^7^, Yingqiu Xie^8^, Xing-Jie Liang^4,5,*^,* *Meng Yu**^3,^*^*^*, Zhiqiang Yu^1,*^*

^1^Department of Laboratory Medicine, Dongguan Key Laboratory of Innovative Molecular Imaging, Dongguan Institute of Clinical Cancer Research, The Tenth Affiliated Hospital, Southern Medical University (Dongguan People’s Hospital), Dongguan 523058, P. R. China

^2^Department of Hepatobiliary, Pancreatic and Splenic Surgery, The Tenth Affiliated Hospital, Southern Medical University (Dongguan People’s Hospital), Dongguan 523058, P. R. China

^3^NMPA Key Laboratory for Research and Evaluation of Drug Metabolism & Guangdong Provincial Key Laboratory of New Drug Screening, School of Pharmaceutical Sciences, Southern Medical University, Guangzhou 510515, P. R. China

^4^CAS Key Laboratory for Biomedical Effects of Nanomaterials and Nanosafety, CAS Center for Excellence in Nanoscience, National Center for Nanoscience and Technology of China, Beijing 100190, P. R. China

^5^University of Chinese Academy of Sciences. Beijing 100049, P. R. China

^6^Institute for Regenerative Medicine, Sechenov University, Moscow 119991, Russia

^7^Department of Experimental Medicine, University of Rome Tor Vergata, Rome 00133, Italy

^8^Department of Biology, School of Sciences and Humanities, Nazarbayev University, Astana 010000, Kazakhstan

**Correspondence**

Xing-Jie Liang, CAS Key Laboratory for Biomedical Effects of Nanomaterials and Nanosafety, CAS Center for Excellence in Nanoscience, National Center for Nanoscience and Technology of China, Beijing 100190, P. R. China

University of Chinese Academy of Sciences. Beijing 100049, P. R. China

E-mail: liangxj@nanoctr.cn

Meng Yu, NMPA Key Laboratory for Research and Evaluation of Drug Metabolism & Guangdong Provincial Key Laboratory of New Drug Screening, School of Pharmaceutical Sciences, Southern Medical University, Guangzhou 510515, P. R. China

E-mail: yumeng999@smu.edu.cn

Zhiqiang Yu, Department of Laboratory Medicine, Dongguan Key Laboratory of Innovative Molecular Imaging, Dongguan Institute of Clinical Cancer Research, The Tenth Affiliated Hospital (Dongguan People’s Hospital), Southern Medical University, Dongguan 523058, P. R. China

1. mail: yuzq@smu.edu.cn

**Funding information**

Natural Science Foundation of Guangdong Province, Grant/Award Number: 2023A1515030291; Science and Technology Program of Guangzhou Grant/Award Number: 202201011130280065; Dongguan Science and Technology of Social Development Program, Grant/Award Number: 20211800905282 and 20231800900362; National Natural Science Foundation of China, Grant/Award Number: 82372115 and 52073139.


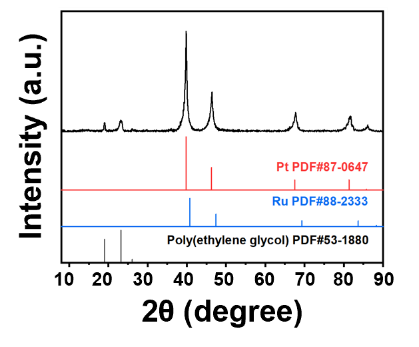


**Figure S1:** The XRD patterns of PR.


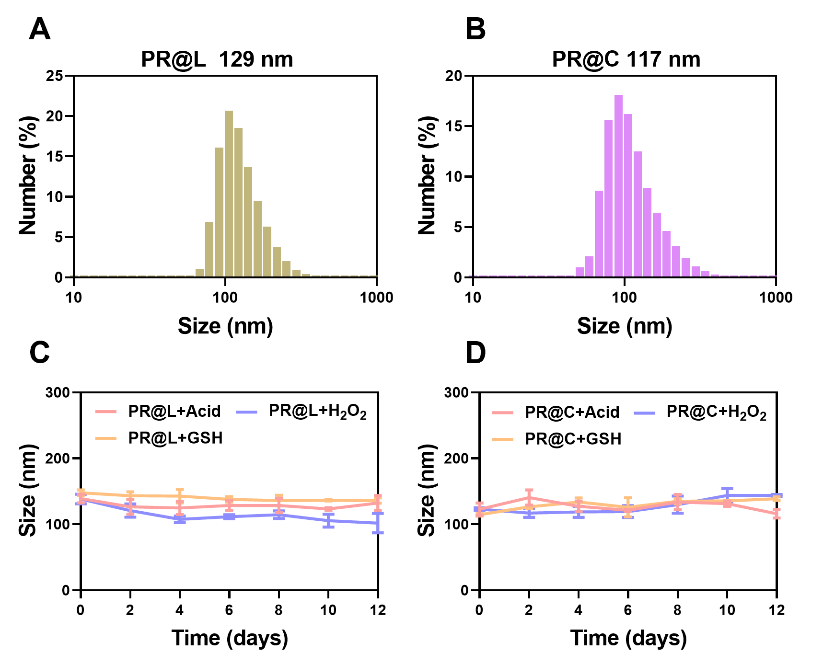


**Figure S2**. The sizes of PR@L (A) and PR@C (B) in the DI water. The long-term stability of PR@L (C) and PR@C (D) in in acidic (pH 6.5), oxidized (100 μM H_2_O_2_) and reduced (10 mM GSH) tumor environment.


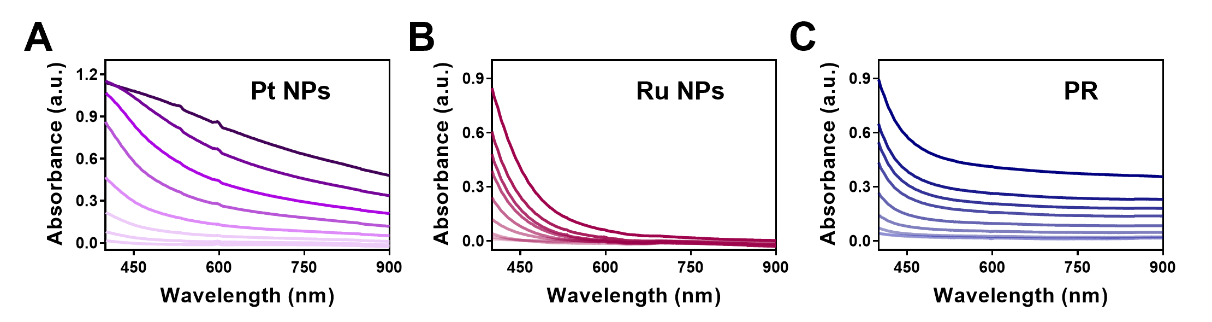


**Figure S3**. UV-vis absorption spectra of Pt NPs, Ru NPs and PR NPs at 2.5, 5, 10, 20, 40, 60, 80, 100 μg·mL^-1^ (Pt) concentrations with gradual deepening color legends.


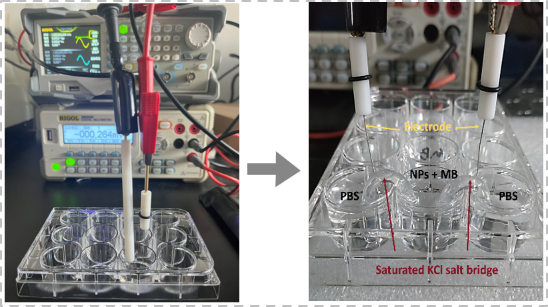


**Figure S4.** The actual picture of EGT platform for *in vitro*.


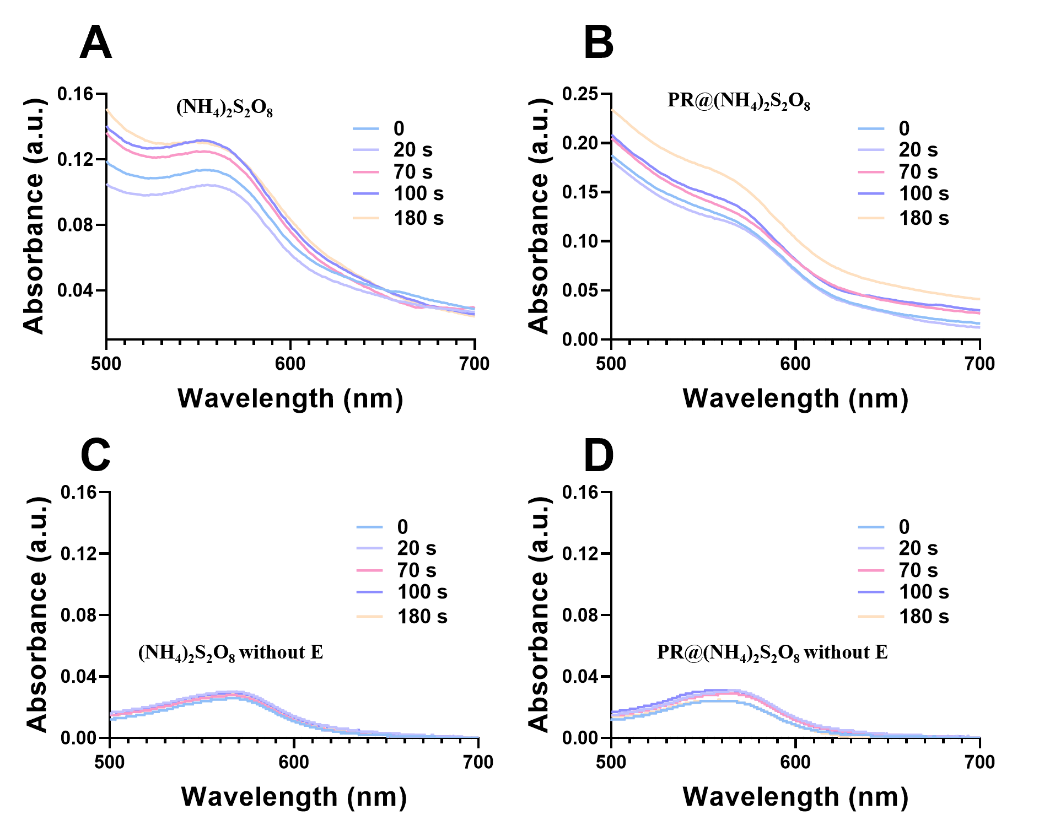


**Figure S5**. UV-vis absorption spectra of sulfur dioxide rapid test kit in the presence of (NH_4_)_2_S_2_O_8_ (A), PR@(NH_4_)_2_S_2_O_8_ (B), (NH_4_)_2_S_2_O_8_ without E (C), PR@(NH_4_)_2_S_2_O_8_ without E (D), maximum absorption wavelength of 536 nm.


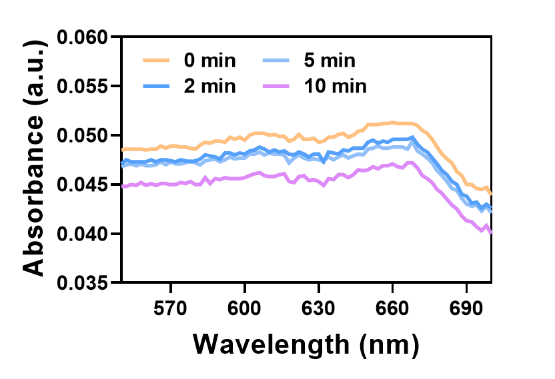


**Figure S6.** UV-Vis absorption spectra of MB solutions degraded by •OH generated by EGT-based electrocatalytic properties in the Ru NPs alone group.


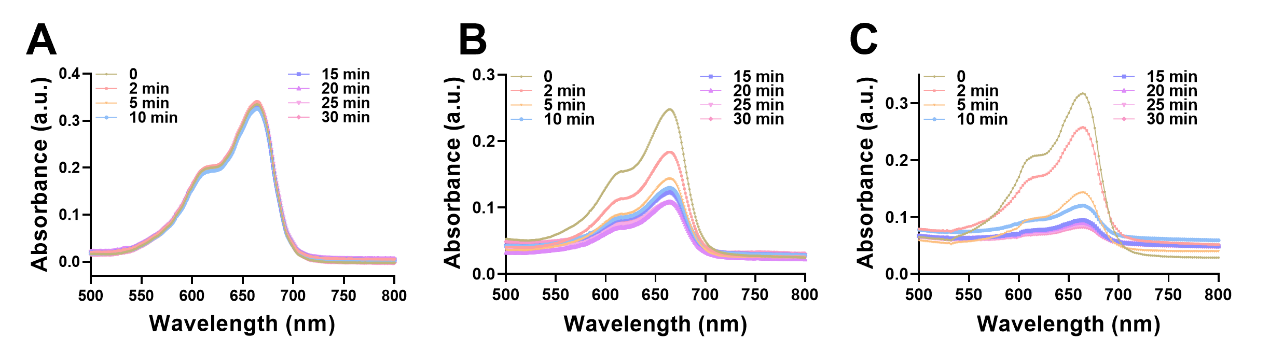


**Figure S7.** UV-Vis absorption spectra of MB solutions degraded by •OH generated by EGT-based electrocatalytic properties in the presence of PBS (A), Pt NPs (B) and PR (C).


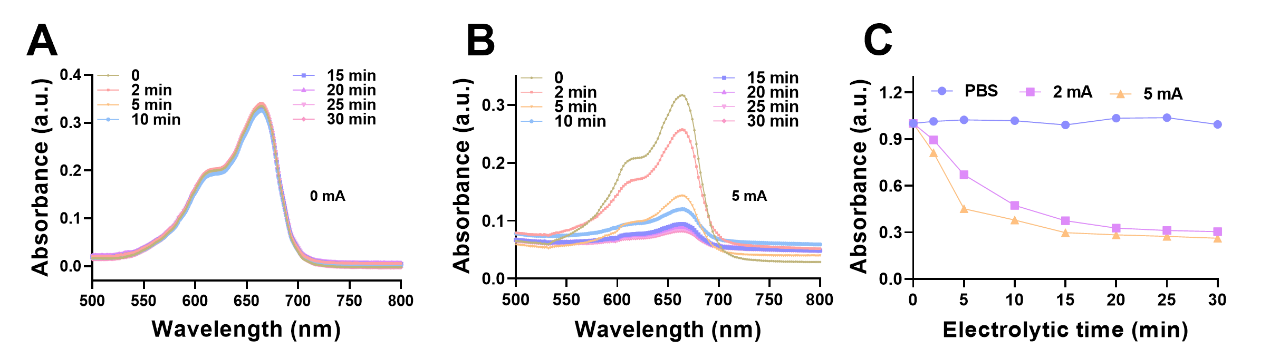


**Figure S8**. Electrocatalytic properties of different EGT formulations at 0 mA (A) and 5 mA (B) current intensities detected by •OH catalyzed MB degradation. MB absorption intensity (at 664 nm) of PR-based EGT electrocatalytic properties at different current intensities 0 mA, 2 mA and 5 mA for 100 s.


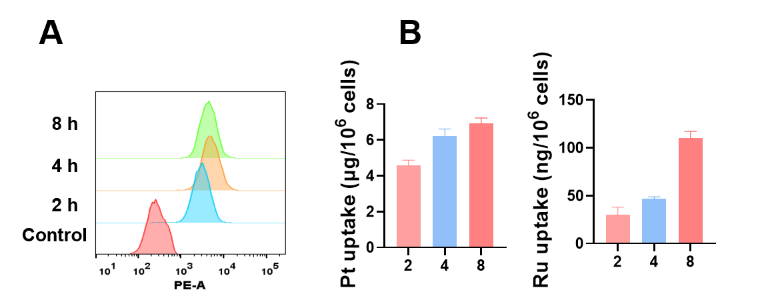


**Figure S9**. (A) Flow cytometer of RhB-labeled PR nano-berries internalized by 4T1 cells at different time. (B) Quantitative analysis of intracellular Pt and Ru content as determined by ICP-MS.


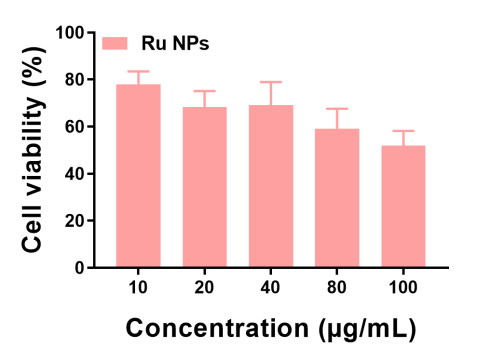


**Figure S10**. Relative cell viabilities of 4T1 cells after incubation with various concentrations Ru NPs for 24 h.


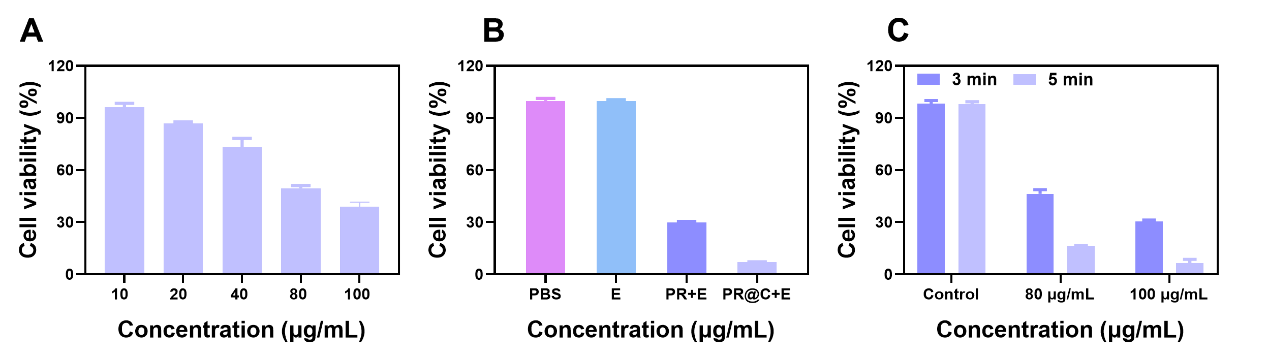


**Figure 11.** (A) Cytotoxicity of 4T1 cells cultured with PR@C at diverse concentrations for 24 h. Relative cytotoxicity of EGT-treated 4T1 cells after incubation with different formulations (at 100 μg·mL^-1^ Pt concentration) (B) and PR@C under various electrical-stimulation time.


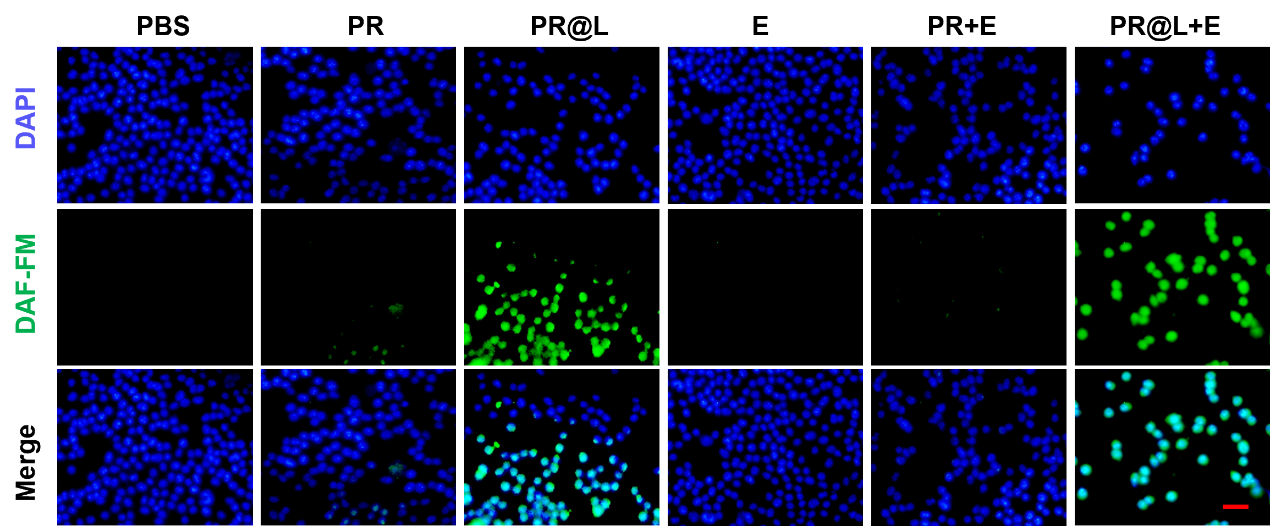


**Figure S12**. Intracellular NO (DAF-FM) production after diverse treatments with or without electrical stimulation monitored by CLSM. Scale bar: 50 µm.


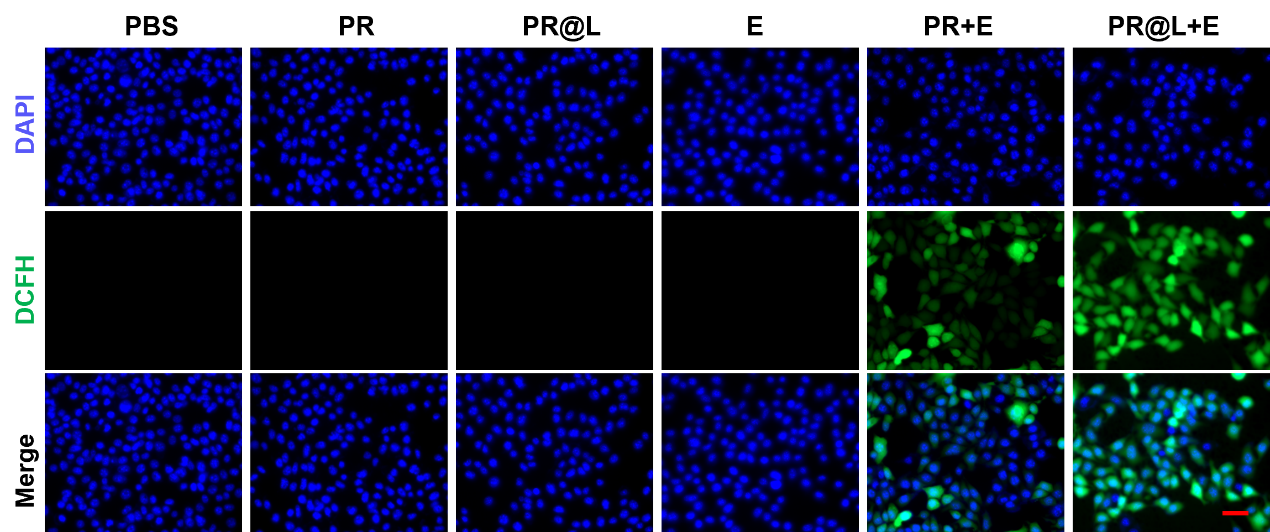


**Figure S13**. Intracellular ROS (DCFH) production after diverse treatments with or without electrical stimulation monitored by CLSM. Scale bar: 50 µm.


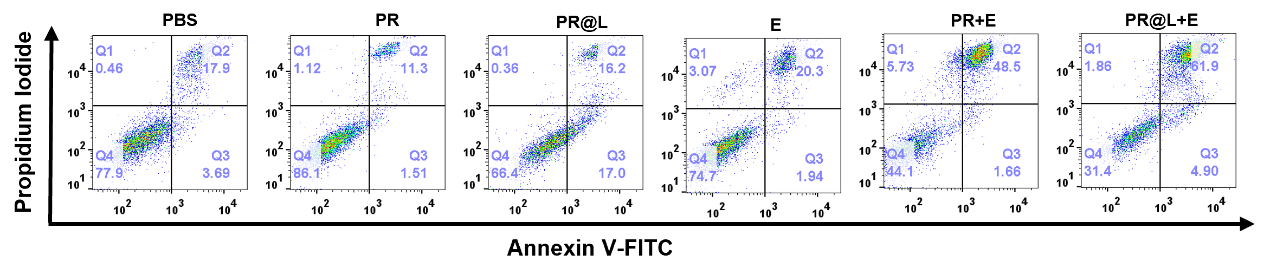


**Figure S14**. Cell apoptosis of EGT-suffered 4T1 cells after different treatments.


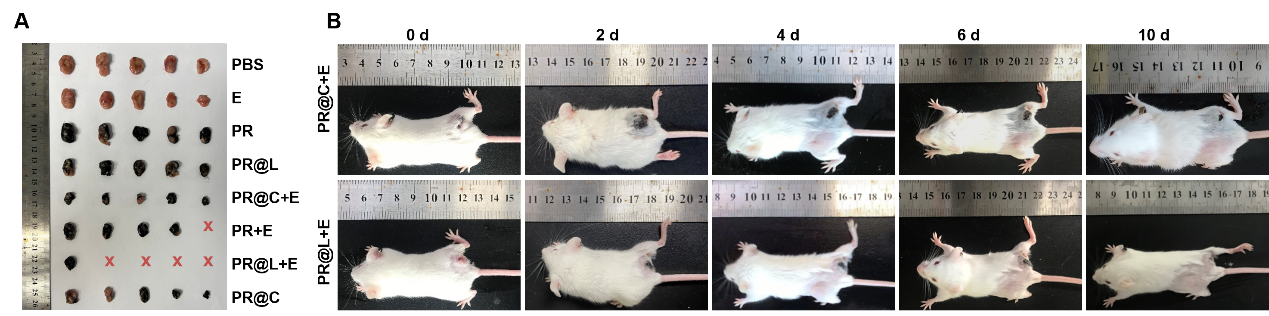


**Figure S15.** (A) The photographs of tumors after treatment. (B) Representative photographs of mice from PR@R-X groups taken at various time points.

**
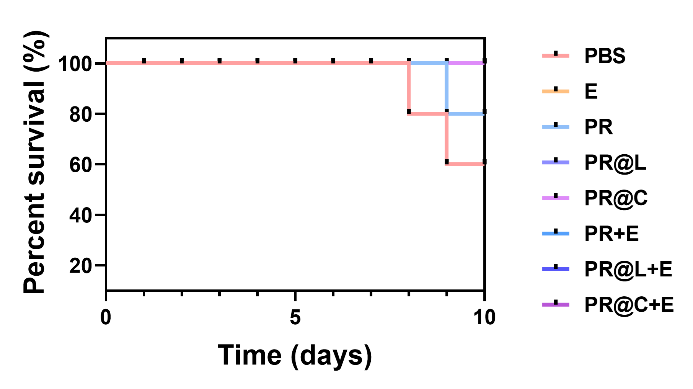
**

**Figure S16.** Survival curves of mice after EGT treatment.


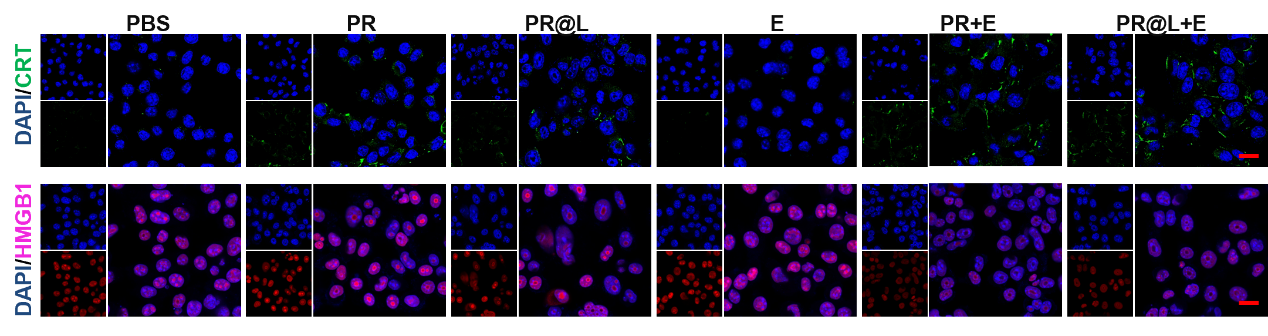


**Figure S17.** CLSM observation of CRT exocytosis to the cell membrane and HMGB1 release from nucleus to cytoplasm *in vitro*. Scale bar: 10 µm.


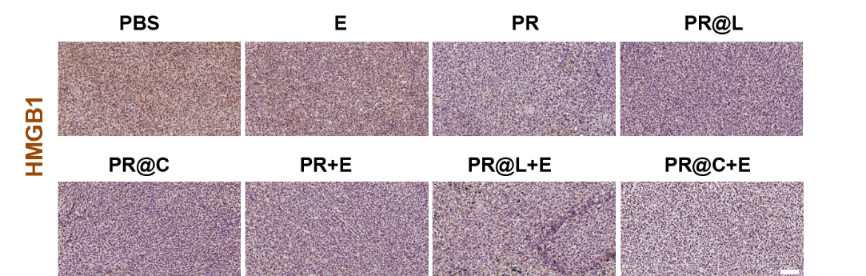


**Figure S18.** Immunohistochemistry analysis for ICD marker (HMGB1 release) in 4T1 tumors from mice after various treatments. Scale bar: 100 µm.


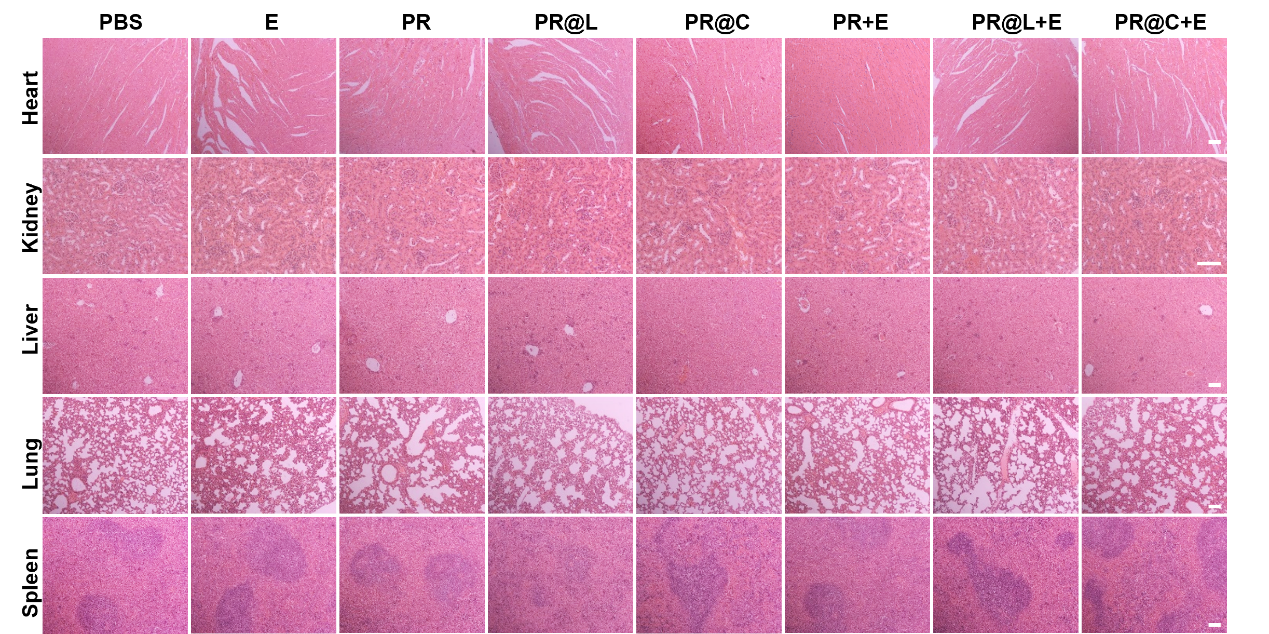


**Figure S19.** *In vivo* pathological study evaluated by H&E analysis of major organs from 4T1 tumor-bearing mice at the end of the treatment (at 10 d), including heart, liver, spleen, lung and kidney. Scale bar: 10 µm.

**
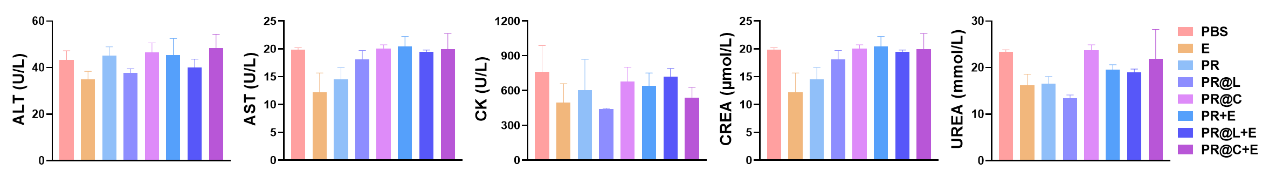
**

**Figure S20.** Biological safety study on serum levels of alanine transaminase (ALT), aspartate transaminase (AST), creatine kinase (CK), creatinine (CR) and urea (UREA) in mice measured at 10 d during the course of treatment. *, P < 0.05, **, P <0.01, and ***, P < 0.001.
